# Impact of Year and Genotype on Benzoxazinoids and Their Microbial Metabolites in the Rhizosphere of Early-Vigour Wheat Genotypes in Southern Australia

**DOI:** 10.3390/plants14010090

**Published:** 2024-12-31

**Authors:** Paul A. Weston, Shahnaj Parvin, Pieter-W. Hendriks, Saliya Gurusinghe, Greg J. Rebetzke, Leslie A. Weston

**Affiliations:** 1 Gulbali Institute for Agriculture, Water and Environment, Charles Sturt University, Wagga Wagga, NSW 2678, Australia; shahnaj.parvin.au@gmail.com (S.P.); sgurusinghe@csu.edu.au (S.G.); leweston@csu.edu.au (L.A.W.); 2 Department of Agricultural Sciences, Lincoln University, Lincoln 7647, Canterbury, New Zealand; pieter-willem.hendriks@lincoln.ac.nz; 3 Agriculture and Food, CSIRO, Canberra, ACT 2601, Australia; greg.rebetzke@csiro.au

**Keywords:** allelopathy, targeted metabolic profiling, UPLC-MS, mass spectrometry, aminophenoxazinones

## Abstract

Wheat (*Triticum aestivum*) is grown on more arable acreage than any other food crop and has been well documented to produce allelochemicals. Wheat allelochemicals include numerous benzoxazinoids and their microbially transformed metabolites that actively suppress growth of weed seedlings. Production and subsequent release of these metabolites by commercial wheat cultivars, however, has not yet been targeted by focussed breeding programmes seeking to develop more competitive crops. Recently, the Commonwealth Scientific and Industrial Organisation (CSIRO), through an extensive recurrent selection programme investment, released numerous early-vigour wheat genotypes for commercial use, but the physiological basis for their improved vigour is under investigation. In the current study, we evaluated several early-vigour genotypes alongside common commercial and heritage wheat cultivars to assess the impact of improved early vigour on the production and release of targeted benzoxazinoids by field-grown wheat roots over a two-year period. Using UPLC coupled with triple quadrupole mass spectrometry (LC-MS QQQ), we quantified common wheat benzoxazinoids and their microbially produced metabolites (aminophenoxazinones) in soil collected from the rhizosphere and rhizoplane of wheat plants over two growing seasons in the Riverina region of New South Wales, Australia. The benzoxazolinone MBOA and several aminophenoxazinones were readily detected in soil samples, but actual soil concentrations differed greatly between years and among genotypes. In contrast to 2019, the concentration of aminophenoxazinones in wheat rhizosphere soil was significantly elevated in 2020, a year receiving adequate rainfall for optimal wheat growth. Aminophenoxazinones were detected in the rhizosphere of early-vigour genotypes and also parental lines exhibiting weed suppression, suggesting that improved early vigour and subsequent weed competitiveness may be related to increased root exudation and production of microbial metabolites in addition to changes in canopy architecture or other root-related early-vigour traits. As previously reported, MBOA was detected frequently in both the rhizoplane and rhizosphere of wheat. Depending on the year and genotype, we also observed enhanced biotransformation of these metabolites to several microbially transformed aminophenoxazinones in the rhizosphere of many of the evaluated genotypes. We are now investigating the role of early-vigour traits, including early canopy closure and biomass accumulation upon improved competitive ability of wheat, which will eventually result in more cost-effective weed management.

## 1. Introduction

Weeds are a critical pest management issue, leading to considerable yield loss for Australian wheat producers. It is estimated that in total, weed infestations cost Australians up to AUD$4.2 billion pa as of 2018 [1]. Yield losses of ~34% are caused by weed infestation in major food crops and are typically higher than losses due to other crop pests [2]. Llewelyn et al. [3] reported the overall cost of weeds to Australian grain growers annually as AUD$3.3 billion, which equates to approximately $150/ha in expenditure and yield losses of ca. 2.8 MT of grain annually.

In recent years, the cost of the cultural and chemical strategies employed to manage weeds in Australia has dramatically increased due to rising costs of petroleum-based fuels, herbicides, labour, and equipment. Continuous use of herbicides and tillage has also led to development of significant weed resistance to multiple herbicide families in cereal production systems over time [4]. Non-herbicide-integrated weed management (IWM) tools are now in high demand to maintain the longevity of new and existing chemistries and reduce the cost of weed management. One such tool of interest includes crop competitiveness with weeds. Recently, we have investigated the competitive ability of cereal crop genotypes to improve weed management via traits that enhance competition for resources including soil moisture, nutrients, and light due to earlier canopy closure [5,6,7,8] and increased early root growth [9]. Competitive crops that can significantly reduce early-season weed growth offer a sustainable management strategy with little to no additional in-crop weed control costs [10,11].

Competitive wheat genotypes have been found to reduce weed numbers by up to 50%, with a corresponding reduction in herbicides needed for their management, by virtue of above-ground competition [8]. Numerous field studies [7,8,12] have shown that combined above- and below-ground crop competition contribute to crop interference with weeds under field conditions. While above-ground competitive ability results from morphological traits that give the crop an advantage in obtaining resources including access to light, much of the below-ground advantage stems from increased root growth or exudation of allelochemicals by roots or leaching from crop residues.

**Figure 1:**
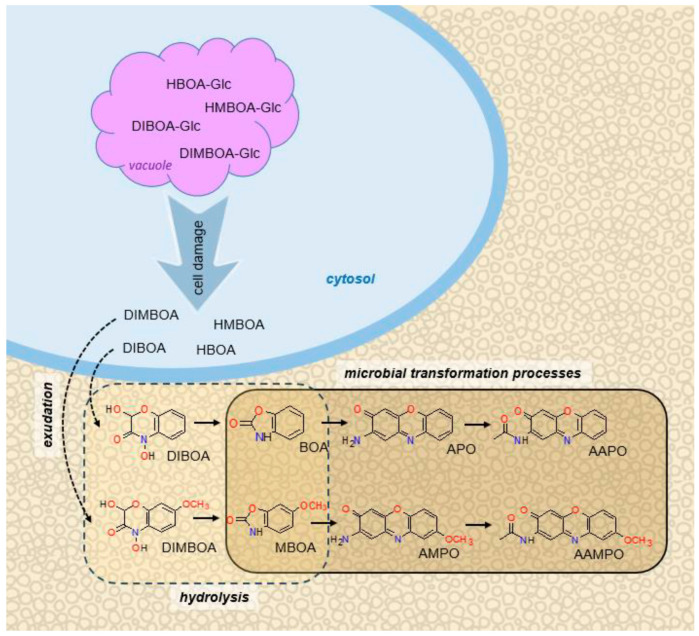
Diagram illustrating the conversion of the hydroxamic acids DIBOA and DIMBOA in the cytosol of wheat root cells by physico-chemical processes to benzoxazolinones (BOA and MBOA) and subsequent microbial transformation to aminophenoxazinones (APO, AAPO, AMPO, and AAMPO) following exudation by wheat roots in the rhizosphere. Adapted from Mwendwa et al. [23].

Wheat plants have been shown to exude the hydroxamic acids DIMBOA (2,4-dihydroxy-7-methoxy-1,4-benzoxazin-3-one) and, to a lesser extent, DIBOA (2,4-dihydroxy-1,4-benzoxazin-3-one), from their roots, which spontaneously convert to the benzoxazolinones MBOA (6-methoxy-3*H*-1,3-benzoxazol-2-one) and BOA (3*H*-1,3-benzoxazol-2-one), respectively, in soil [10,13] (Figure 1). Under the appropriate field conditions, a range of fungi and bacteria can transform MBOA to the aminophenoxazinones 2-amino-7-methoxy-phenoxazin-3-one (AMPO) and 2-acetylamino-7-methoxy-phenoxazin-3-one (AAMPO) [13,14,15,16,17,18,19,20] and BOA to the aminophenoxazinones 2-amino-3H-phenoxazin-3-one (APO) and 2-acetylamino-phenoxazin-3-one (AAPO) [20,21]. Benzoxazilinones generally possess herbicidal properties to a limited extent and are fairly short-lived in soil, but aminophenoxazinones, particularly APO and AMPO, are potent herbicides and can persist in soils for weeks or months [20].

Suppression of weeds via allelopathic agents, including benzoxazolinones and their phenoxazinone biotransformation products associated with wheat and closely related cereal crops, has been well-documented both in Europe [19] and North America [22] and, most recently, in Australia [23]. In natural plant communities, early vigour, defined as more rapid leaf area development through wide leaves and greater biomass at stem elongation [24], is a common mechanism for plant-to-plant competition [25]. Introducing early-vigour traits into wheat cultivars has resulted in a more weed-competitive crop [26]. In wheat, traits relating to leaf size and width, leaf area, and rate of growth or biomass accumulation, as well as root growth, root architecture, and root exudation, vary between genotypes and have all been linked to greater weed-suppressive ability [9,11,23,27,28]. Moreover, Mwendwa and co-workers determined that early-vigour wheat genotypes also produced allelochemicals at ecologically relevant concentrations under dryland cropping conditions in Australia [23].

Research by the Rebetzke team at the Australian Commonwealth Science and Industry Research Organisation (CSIRO) over the past 20 years has successfully utilised recurrent selection to increase early shoot vigour in wheat germplasm [29]. The top-cross of this enhanced vigour material into commercial cultivars resulted in enhanced early vigour through modified canopy architectural traits and reduced germination and establishment of weeds [9]. The specific mechanisms associated with early-vigour wheat and weed suppression are being further investigated, and recent research has shown enhanced root hair length is also associated with early vigour in some genotypes. The research reported herein addresses the potential for a chemical interference mechanism of weed suppression in wheat by quantification of exuded wheat metabolites (i.e., benzoxazinoids) and their potently active microbial metabolites (i.e., aminophenoxazinones) in the rhizosphere of selected wheat genotypes and compares their presence in commercial cultivars with novel release of early-vigour selections under field conditions over two growing seasons.

## 2. Results

**Figure 2:**
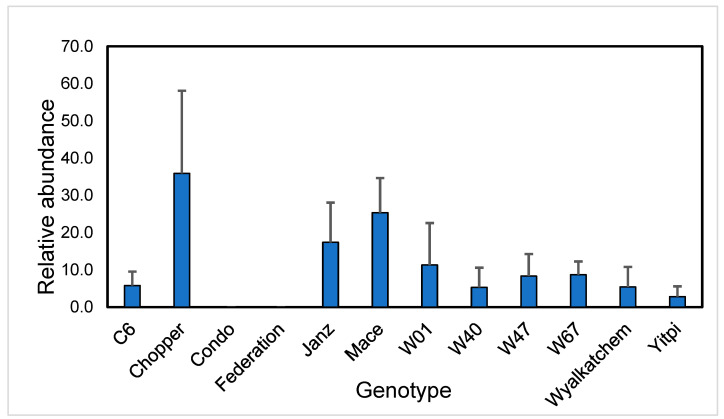
Relative abundance of MBOA in root rhizoplane of wheat cultivars grown in field plots at mid-tillering in 2020. Values are relative abundance adjusted for weight of root samples (+standard error).

The UPLC-MS method developed for separation of metabolites was able to detect and quantify both benzoxazolinones and phenoxazinones in soil and the wheat rhizoplane with increased sensitivity and precision in contrast to previous studies. Based on previous experimentation performed by Mwenda et al. [23] characterising secondary metabolites exuded by wheat, we focused on the presence of two key benzoxazolinones (MBOA and BOA) and the microbially transformed aminophenoxazinones APO, AAPO and AMPO. MBOA was also detected in the rhizoplane of all genotypes, except Condo and Federation, and was notably more abundant in the rhizoplane of Chopper (Figure 2), although the high variability in these values meant these differences were not statistically different. BOA was not detected in any rhizoplane samples but was detected in the soil rhizosphere. We note that the lack of MBOA detected in the rhizosphere of Condo and Federation may not have resulted from lack of production but rather the complete transformation of parent molecule to aminophenoxazinones.

**Figure 3:**
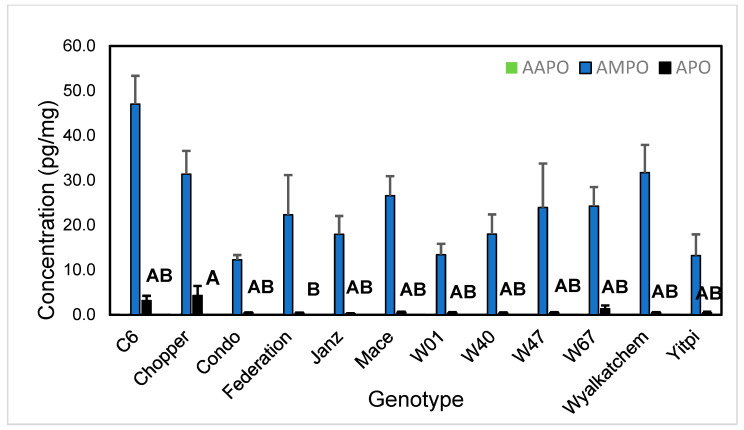
Abundance of aminophenoxazinones in root rhizoplane of wheat cultivars grown in field plots on 1 July 2020 (early tillering). Values are mean concentration per mg of root tissue (+standard error). Letters adjacent to bars indicate significant differences in abundance of APO (bars accompanied by the same letter are not statistically different as determined by Tukey’s honestly significant difference). Bars for AAPO and AMPO have no accompanying letters because levels of these metabolites did not differ among genotypes, and AAPO ws not detected.

The phenoxazinone APO was readily detected at a concentration as low as 100 pg/mL, but the limit of quantitation (LOQ) was ca. 1 ng/mL; below this concentration, the detector response was no longer linear. The detector response to APO was linear up to 1 ug/mL, the highest concentration used for constructing dose/response calibration curves. In the root rhizoplane, AMPO was the predominant aminophenoxazinone detected, while APO and AAPO were at trace levels or virtually absent from root samples (Figure 3). Levels of the aminophenoxazinones were not significantly different among genotypes, except for APO, which was significantly higher in Chopper (a triticale cultivar) than in Federation, as determined by Tukey’s honestly significant difference test at an experiment-wise error rate of *p* < 0.05.

**Figure 4:**
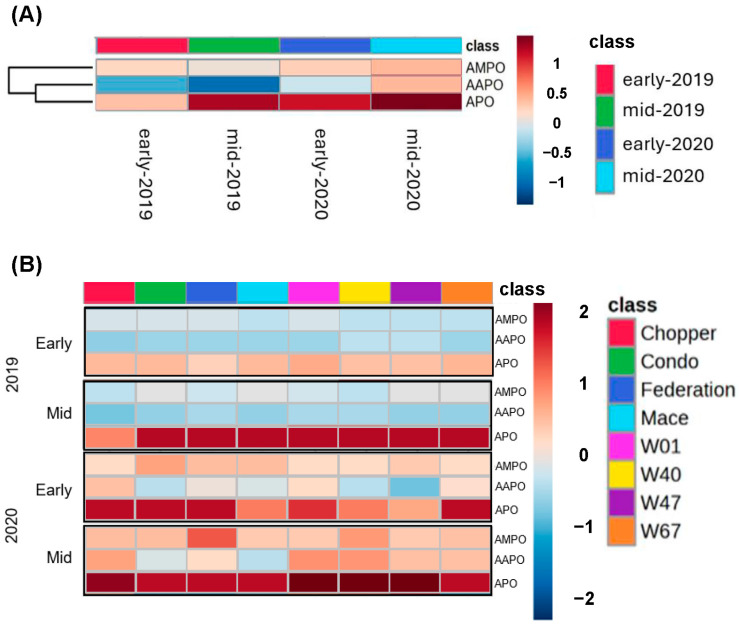
Heat maps showing differential abundance of aminophenoxazinones in the rhizosphere among genotypes and growth stage for wheat genotypes common to both years of the study. Levels of abundance, from high to low, are coloured from dark red to dark blue. (**A**) Relative abundance of the three aminophenoxazinones averaged over all genotypes for the two stages of plant growth (early and mid-tillering) and year (2019 and 2020). (**B**) Relative abundance of aminophenoxazinones at early and mid-tillering across eight genotypes common to both years of the study. Data for both heat maps were log_10_-transformed.

Interestingly, neither BOA nor MBOA was detected in rhizosphere samples in 2019 or 2020. However, aminophenoxazinones were detected in the rhizosphere and varied depending on the year and crop maturity stage (Figure 4), suggesting that microbial transformation of parent molecules was complete or nearly so past the point of detection. Overall, the abundance of these biotransformed metabolites was higher in 2020 than in 2019 and was higher at the mid-tillering than at the early tillering stage in both years (Figure 4A). In contrast to the rhizoplane samples collected on the root surface, where AMPO predominated, APO was the most abundant aminophenoxazinone in the rhizosphere samples. There was limited variability in the levels of APO among genotypes, but a few clear patterns were observed. At the mid-tillering stage in 2019, rhizosphere soil from the triticale cv. Chopper plots contained lower levels of APO than wheat genotypes, but in 2020, at mid-tillering, Chopper was among those genotypes containing the highest levels of APO in the rhizosphere. The early-vigour genotypes W01, W47, and W67 exhibited the highest levels of APO in rhizosphere soil at mid-tillering in 2020.

**Figure 5:**
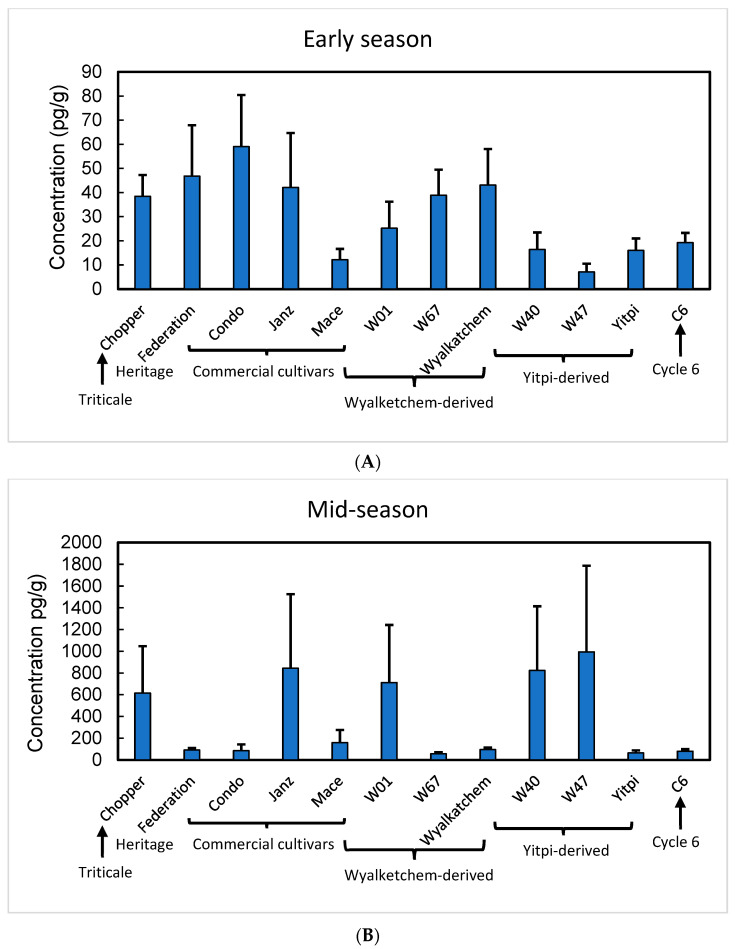
Abundance of total aminophenoxazinones in rhizosphere samples at (**A**) early tillering and (**B**) mid-season from soil of wheat cultivars grown in field plots in 2020. Note the difference in scale on the y-axis between early- and mid-season samples. Error bars are one standard error.

In 2020, the experimental treatments further included cv. Wyalkatchem, from which W01 and W67 were derived, and Yitpi, the commercial parent line from which W40 and W47 were derived. Additionally, an experimental accession derived from sixth cycle of the recurrent selection for shoot vigour (C6) was included owing to its early vigour and high biomass. Compared to the parental wheat cultivars, most of the samples from experimental cultivars showed similar abundance of total aminophenoxazinones at early tillering (Figure 5A). By mid-tillering, however, the experimental lines with the exception of W67 had accumulated substantially higher levels of total aminophenoxazinones in the rhizosphere than the parental material (Wyalkatchem and Yitpi) (Figure 5). Total aminophenoxazinones were significantly higher at midseason compared to early season in both 2019 (*F*_1,95_ = 84.1, *p* < 0.0001, and 2020 (*F*_1,95_ = 57.4, *p* < 0.0001), although there were no statistical differences between genotypes because of large genotype-to-genotype variability.

## 3. Discussion

The wheat genotypes evaluated over two years under field conditions varied greatly in the level of aminophenoxazinones detected from soil samples collected over time. Interestingly, these bioactive metabolites were apparently most abundant in the rhizosphere of several advanced early-vigour wheat genotypes selected by recurrent selection in specific breeding lines (e.g., W01, W40, and W47) and lowest in the rhizosphere of heritage and commercial cultivars, with the exception of Janz, a commercial cultivar which was comparable to early-vigour genotypes. Interestingly, in the early 2000s, Wu et al. [30] studied the allelopathic potential of wheat and discovered when evaluating root exudates of various wheat genotypes that the presence of phenolic acids and DIMBOA did not fully account for the strong inhibitory potential of key genotypes. Near isogenic lines derived from Hartog (Pavon-background) and Janz (Condor-background) were further screened in order to investigate the genetic control of wheat allelopathy with *L. rigidum* as a test species [31]. Of the two parents, Hartog was observed to be weakly allelopathic, while Janz was observed to be strongly allelopathic in laboratory assays. Given the well-documented phytotoxicity of aminophenoxazinones [32,33,34], the observed increase in abundance of these biotransformed allelochemicals may help to explain the increased weed-suppressive potential of several early-vigour genotypes, as well as Janz, as demonstrated in concurrent field evaluations of naturally emerging weeds in the same research plots [9,35]. Moreover, these genotypes might produce improved yields in the absence of herbicide application as a result of reduced competition from weeds [9].

**Figure 6:**
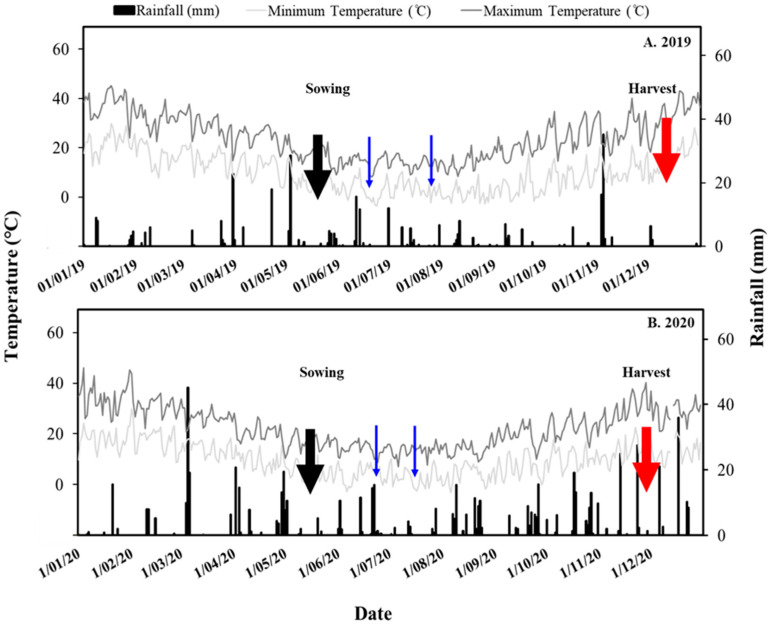
Seasonal rainfall (black bars) and maximum and minimum temperatures (grey lines) at the Wagga Wagga site for 2019 (**A**) and 2020 (**B**). Arrows denote the sowing (black arrows) and the harvest (red arrows) of winter crops. Sampling events are indicated by blue arrows in both years.

The greater abundance of aminophenoxazinones observed in rhizosphere samples in 2020 than in 2019 is likely the result of greater root exudation of hydroxamic acids (the parent molecules of benzoxazolinones, which in turn are the precursors of aminophenoxazinones) or possibly the enhanced microbial activity and thus greater rates of biotransformation of benzoxalinones in moist soils in 2020 compared to the exceptionally dry soils encountered in 2019 (Figure 6). The 2020 season was marked by abundant rainfall and improved soil moisture status. It is certainly plausible that improved root development under more suitable growing conditions in 2020 may have led to an increase in the abundance of below-ground organic carbon, which favours rapid microbial metabolism of benzoxazolinones [35]. In addition, Hendriks et al. [8] found that early-vigour wheat lines demonstrated enhanced growth and density of root hairs under controlled conditions. Enhanced root hair development may also be associated with increased exudation of hydroxamic acids and microbial transformation of benzoxazolinones to the more phytotoxic aminophenoxazinones.

Greater abundance of aminophenoxazinones was also noted in soil samples collected at later stages of crop development in contrast to earlier stages. Mwendwa et al. [23] found little change in MBOA levels over time in the root rhizoplane of wheat cultivars grown in similar soils and locale to those in our study. However, in Mwendwa’s study, field samples were first collected and assessed at a growth stage more advanced than mid-tillering, while older plants closer to harvest exhibited reduced levels of MBOA and aminophenoxazinones in the wheat rhizoplane. In the current study, wheat plants were sampled in both early and mid-season and showed increasing production of aminophenoxazinones by mid-season (at the same point when Mwendwa et al. first initiated sampling). Although we observed variability in environmental conditions between years, particularly with regard to rainfall and temperature, both sampling time and the local environment are likely important factors accounting for observed differences (Figure 6).

The virtual absence of MBOA, which is known to be an inhibitor of auxin-dependent growth [36], in the rhizoplane samples of Federation and Condo in our study, compared to the high levels of MBOA reported for these cultivars by Mwendwa et al., was a significant difference noted between our results and those of Mwendwa et al. [23]. Differences in weather conditions may account for differences in root growth and root exudation, and it is feasible that the DIMBOA exuded by the roots of Federation was also very rapidly and completely converted to AMPO by the microbial community in the rhizosphere and rhizoplane; the levels of AMPO in the rhizoplane of Federation roots were similar to the levels observed for the other genotypes in our study, supporting this hypothesis. It is also conceivable that the rhizoplane of Federation and Condo support more diverse and active microbial communities than the other genotypes evaluated in our study, possibly due to differences in root exudation and rapid microbial transfer, which could lead to more rapid conversion of MBOA to aminophenoxazinones.

We also noted an absence of BOA in any of the rhizoplane samples analysed in the current study and the predominance of APO over AMPO and AAPO in soil samples, again in contrast to the results observed by Mwendwa et al. [23]. Although DIMBOA, the parent molecule of MBOA, is known to be the predominant benzoxazinoid exuded by wheat [8], DIBOA (the parent molecule of BOA) is also exuded to a lesser extent. Mwendwa et al. [23] readily found BOA in the roots and rhizoplane of all wheat cultivars evaluated in their study; the absence of BOA in the rhizoplane of wheat genotypes evaluated in 2020 in our study and the predominance of APO (the breakdown product of BOA) in soil samples suggests that microbial activity in the rhizoplane of wheat plants in our study was significantly different and potentially more active compared to that present during Mwendwa’s study and, in 2020, possibly encouraged full conversion to APO from the parent molecule DIBOA.

The presence of MBOA and the absence of BOA in the root rhizoplane of wheat samples were not unexpected, as wheat is known to produce and exude mainly DIMBOA, which is spontaneously hydrolysed to MBOA in soil [10]. The predominance of AMPO in the root rhizoplane was likewise expected because soil bacteria and fungi are known to convert MBOA to AMPO after conversion of DIMBOA to MBOA [13,14,15,16,17,18]. The predominance of APO in soil samples is more difficult to explain because APO arises from microbial transformation of BOA, which was not detected in any of our rhizoplane or rhizosphere samples. One possible explanation is that DIBOA (the parent molecule of BOA) was exuded but very rapidly and completely transformed to APO by soil microbes.

While wheat roots are commonly observed to exude DIMBOA, they are also known to produce DIBOA depending on the cultivar and environmental conditions encountered. For example, in research performed by Hussain et al. 2022 [32], weeds were co-germinated with the wheat cultivar Ursita for 10 days. In this case, root exudates from Ursita reduced the seedling growth of annual ryegrass, *Lolium rigidum*, by 29–60%, depending on co-culture conditions of planting density. Weed pressure caused significant increase in the production of phenolic acids (vanillic, ferulic, syringic, and *p*-coumaric acids) in shoot tissues and soil abundance of benzoxazinoids, in particular DIMBOA, MBOA, HBOA (2-hydroxy-7-methoxy-1,4-benzoxazin-3-one), and HMBOA (2-hydroxy-1,4-benzoxazin3-one), under laboratory conditions. It is also conceivable that interactions of field-grown wheat with numerous weeds encountered in 2020 resulted in increased production and exudation of DIBOA rather than DIMBOA.

Our findings suggest that enhanced production of allelochemicals, specifically benzoxazolinones, by early-vigour lines and other suppressive cultivars such as the historic wheat Federation and their biotransformation to phenoxazinones may be responsible in part for enhanced competitiveness of wheat. These findings, coupled with previous work [9,10,27], highlight the fact that selection for shoot vigour has pleiotropic effects on key traits of wheat (e.g., allelopathic potential and crop architecture). Indeed, those early-vigour lines and cultivars exhibiting rapid canopy closure have repeatedly demonstrated superior weed suppression [5,6,7,8,27]. Our findings align with those of Bertholdsson [12], who suggested that both early vigour and allelopathy were desirable traits to improve the competitiveness of crops against weeds. Although it is sometimes difficult to ascertain the relative contribution of morphological attributes vs. allelochemicals toward crop competitiveness, as clearly summarised by Mahé et al. [37], it is clear that the ability of crop plants to exude allelochemicals at biologically relevant concentrations under field conditions [34] constitutes an important dimension of competitiveness vis-a-vis weeds.

However, a key challenge remains to screen, improve, and characterise genotypes offering consistent production and release of allelochemicals under field conditions while developing agronomic practices to support optimal establishment and rapid canopy closure. Genotypes exhibiting enhanced root growth and root exudation plus early vigour may eventually prove to be important in achieving effective weed management solutions, especially if multiple modes of weed suppression are on offer. In order to facilitate a more holistic approach to breeding and selection of competitive wheat cultivars, it is important to consider both chemical and morphological traits associated with weed suppression. In this case, despite the fact that wheat root architecture can be challenging to examine, considerable differences were noted in both controlled environment and field assays in terms of root architecture, root hair formation, root exudation, and presence of bioactive rhizosphere metabolites in early-vigour genotypes when compared to commercial or historic cultivars. Our findings suggest that thorough screening of belowground traits may also drive the selection of highly suppressive wheat genotypes.

## 4. Materials and Methods

### 4.1. Site Selection and Experimental Design

Two field experiments were carried out during the field-growing seasons of 2019 and 2020 at the EH Graham Centre in Wagga Wagga, NSW (35°03′ S, 147°36′ E; 227 m altitude). The soil at the trial site was classified as a fine red clay–loam kandosol. The pH of the topsoil (0–10 cm) was 6.4, and beyond that depth, the pH was low (4.9).

The long-term average annual rainfall of the trial location was 577 mm (Australian Bureau of Meteorology, www.bom.gov.au, accessed on 14 November 2021). Due to the prevailing El Niño condition during the 2019 crop-growing season, high temperatures coupled with lower precipitation shortened the duration of crop growth period. In contrast, 2020 was a very wet year. Rainfall during the growing season was 205 and 446 mm in 2019 and 2020, respectively, compared with the long-term average of 364 mm. Temperature and rainfall patterns in Wagga Wagga for 2019 and 2020 are shown in Figure 6.

In both years, wheat genotypes were sown with five replications in a randomised complete block design. The plots were 10 m long × 1.8 m wide (8 rows at 20 cm row spacing). Sowing rates for treatments were calculated with the aim of establishing 150 plants/m^2^. The plots were sown in mid to late May. Di-ammonium phosphate (DAP) was applied at the time of sowing, whereas supplemental urea (Incitec PivotTM Fertilisers, Southbank, VIC, Australia) was supplied as needed following soil testing. Before sowing, all existing established weeds were controlled using a non-selective herbicide (glyphosate, Weedmaster^®^DST^®^ 470 g/L glyphosate, Nufarm Australia, MEL, Australia) at 960 g/ha.

### 4.2. Plant Material

In 2019, ten genotypes were assessed: five early-vigour wheat lines (W01, W32, W40, W47, and W67), two commercial wheat cultivars (Condo and Mace), two heritage cultivars (Federation and Ford), and triticale (cv Chopper). The 2020 field trial assessed the following genotypes: five early-vigour wheat lines (C6, W01, W40, W47, and W67), five commercial wheat cultivars (Condo, Janz, Mace, Wyalkatchem, and Yitpi), one heritage cultivar (Federation), and triticale (cv Chopper). Common to both years were W01, W40, W47, W67, Condo, Mace, Federation, and Chopper.

### 4.3. Root and Soil Sampling

Samples consisted of rhizoplane soil or bulk soil taken from two subplots per plot. For each subplot, four samples were taken along a diagonal transect to a depth of 10 cm, which were then combined to form one sample for each subplot. These samples were later merged to create one composite sample for each plot in each of six replicates in 2019 and five replicates in 2020. For each year, soil samples were collected at two stages of crop development designated “early” (25 June 2019 and 1 July 2020) and “mid” (31 July 2019 and 16 July 2020).

Root samples were collected only once during the experiment (1 July 2020). Plants were uprooted and gently shaken to remove the soil adhering to the roots. After removing the shoots, roots were kept in a ziplock bag and stored at −20 °C until later analysis.

### 4.4. Soil and Root Extraction Procedures

The extraction procedures for soil and root rhizoplane samples were based on those of Fomsgaard et al. [10]. Soil samples were removed from the freezer and dried at 40 °C for 72 h. Dried soil (5 g) was weighed and transferred to 250 mL Erlenmeyer flasks along with 50 mL of extracting solvent (99.5:0.5 methanol: acetic acid). The flasks were shaken on a rotary shaker (Ratek Instruments, Boronia, VIC, Australia) at 125 rpm for 120 min, after which the soil suspension was filtered through Whatman No. 1 filter paper and air-dried in a fume cupboard for 48–72 h. The residue was resuspended in 10 mL methanol and transferred to 20 mL scintillation vials. Prior to analysis via LC-MS, samples were filtered through 0.22 μm polytetrafluoroethylene (PTFE) syringe filters (Captiva Econofilter, Agilent Technologies, Santa Clara, CA, USA) and stored at 4 °C.

Root rhizoplane samples (5–10 g) were weighed and transferred to 250 mL Erlenmeyer flasks with 50 mL of extracting solvent and shaken on a rotary shaker as described above. The suspension was filtered through Whatman No. 1 filter paper, air-dried and reconstituted in methanol, and filtered as before. The extracted root tissue was rinsed under a stream of water, placed in a Petri dish, and allowed to air dry for 72 h, after which the root dry weight was recorded.

### 4.5. Quantitation of Secondary Metabolites and Microbial Transformation Products from Wheat

Metabolic profiling of the extracts for targeted secondary metabolites was performed using high-pressure liquid chromatography interfaced with a triple quadrupole mass spectrometer (LC-MS QQQ) using similar methods as described in Mwendwa et al. [8]. Separation of analytes was achieved using HPLC (Agilent 1290 Infinity UHPLC equipped with binary pump, diode array detector, degasser, and temperature-controlled column @ 25 °C) on a C18 analytical column (Poroshell 120 SB-C18, 2.1 mm × 100 mm, 2.7 µm particle size) (Agilent, Santa Clara, CA, USA) preceded by a guard column of similar (but not identical) composition (Poroshell 120 Bonus-RP, 2.1 mm, 2.7 µm) (Agilent, Santa Clara, CA, USA). Solvent A was municipal water purified with Milli-Q filtration system and solvent B was 95% acetonitrile (Source, location): 5% Milli-Q water; both solvent A and B were amended with formic acid (Merck, Baywater, VIC, Australia) at a rate of 0.1% *v*/*v*. The mobile phase composition started at 25% solvent B for 1 min, then increased to 80% solvent B from 1 to 13 min, followed by an increase to 100% B over the next minute. The mobile phase remained at 100% B for 3 min, then was dropped to the starting conditions (75:25 solvent A:B) and held for 7 min to allow the column to re-equilibrate.

Benzoxazolinones (BOA and MBOA) and their microbial transformation products (the aminophenoxazinones APO, AAPO, and AMPO) (Table 1) were quantified using an Agilent 6410 triple quadrupole mass spectrometer equipped with an Agilent Dual Jet Stream electrospray ionisation source. The instrument was operated in positive and negative ion modes at the appropriate time intervals to detect the analytes of interest. The machine was run in negative ion mode for the first 7 min of each separation to detect BOA and MBOA, if present, and in positive ion mode for the rest of each run to detect APO, AAPO, and AMPO. Dynamic multiple-reaction monitoring (MRM) was used to detect and quantify the analytes of interest; the parameters used for the individual compounds are shown in Table 2. The capillary voltage was set at +3500 V. Nitrogen was used as the drying gas at a flow of 9 L min^−1^, and nebulizer pressure was 35 psi. Sheath gas flow and temperature were 10 L min^−1^ and 250 °C, respectively.

Every 15 samples, a quality control sample and blank were analysed to confirm system performance. An injection of a mixed standard of APO, AAPO, and AMPO (provided by I. Fomsgaard, Aarhus University, Denmark) in equal proportions was injected periodically to confirm retention time consistency and uniformity of detector response. In addition, a dilution series of APOMerck, Baywater, VIC, Australia) was run at the beginning and end of each set of samples in order to quantify the abundance of the aminophenoxazinones. The abundance of benzoxazolinones was expressed in units of detector response as purified standards of BOA and MBOA were not available. APO could be quantified directly by comparison of detector response to the dose/response curve constructed for this compound from the dilution series, which ranged in concentration from 100 pg/mL up to 1 μg/mL in log steps. The quantities of AAPO and AMPO available were insufficient to create dilution series for these compounds, so AAPO and AMPO were quantitiated using the response curve for APO adjusted by the sensitivity factors of these compounds relative to APO (sensitivity ratio of AAPO:APO = 5.04, AMPO:APO = 0.24). The abundance of the aminophenoxazinones was calculated in units of picograms per gram of soil (for soil samples) and picograms per gram of root tissue (for root samples).

### 4.6. Data Analysis

Statistical testing of MBOA and aminophenoxazinones was conducted using factorial analysis of variance (ANOVA) after transforming data with log_10_ (x + 1) (Statistix 10.1, Analytical Software, Tallahassee, FL, USA). After transforming the data, values for AAPO, AMPO, and APO fit the assumptions required for ANOVA (non-additivity, normality, and homogeneity of variances), as determined by Tukey’s one degree of freedom, Shapiro–Wilk, and Levene’s tests, respectively. Values for MBOA could not be made to fit these assumptions regardless of the transformation used. Heat maps were generated with MetaboAnalyst 6.0 online (https://new.metaboanalyst.ca/MetaboAnalyst/home.xhtml, accessed on 20 December 2024) after transforming each analyte with log_10_ transformation. Parameters used for heat map generation by MetaboAnalyst are as follows: Data scaling—none; Distance measure—Euclidian; Clustering algorithm—Ward.

## Figures and Tables

**Table 1:** Systematic names and molecular properties of major benzoxazolinones exuded by wheat roots and their microbial transformation products.

Compound	Systematic Name	Molecular Formula	Monoisotopic Mass
BOA	2-benzoxazolinone	C_7_H_5_NO_2_	135.1201
MBOA	6-methoxy-2-benzoxazolinone	C_8_H_7_NO_3_	165.1461
APO	2-amino-3-H-phenoxazin-3-one	C_12_H_8_N_2_O_2_	212.2080
AMPO	9-methoxy-2-amino-3-H-phenoxazin-3-one	C_13_H_10_N_2_O_3_	242.2340
AAPO	2-acetylamino-3-H-phenoxazin-3-one	C_14_H_10_N_2_O_3_	254.2450
AAMPO ^1^	2-acetylamino-9-methoxy-2-amino-3-H-phenoxazin-3-one	C_15_H_12_N_2_O_4_	284.2710

^1^ Compound not quantified in this study.

**Table 2:** Parameters used for multiple reaction mode monitoring of wheat metabolites in soil.

Compound	Precursor Ion	Product Ion	Retention Time (min)	Retention Time Window	Fragmentor Voltage	Collision Energy	Acceleration Voltage	Polarity
BOA	134	42	4.5	1.2	135	20	5	negative
MBOA	164	149	5.3	1.2	135	20	5	negative
APO	213	185	9.2	1.2	130	24	5	positive
AMPO	243	228	10.1	1.2	135	20	5	positive
AAPO	255	213	10.8	1.2	135	20	5	positive

## Data Availability

Data is held on the cloud at Charles Sturt University for a period of 7 or more years.
